# Mucinous Micropapillary Carcinoma of Breast in a Developing World Setting: Case Series With Clinicopathological Insights and Survival Analysis From a Tertiary Care Hospital

**DOI:** 10.1155/crip/5573148

**Published:** 2026-03-08

**Authors:** Saba Anjum, Mehwish Mooghal, Naila Kayani, Abida Sattar, Lubna Vohra, Sana Zeeshan, Romana Idress

**Affiliations:** ^1^ Department of Pathology, Zia Uddin University Hospital, Karachi, Pakistan; ^2^ Department of Breast Surgery, Aga Khan University Hospital, Karachi, Pakistan, aku.edu; ^3^ Department of Pathology and Laboratory Medicine, Aga Khan University Hospital, Karachi, Pakistan, aku.edu

**Keywords:** breast cancer, case reports, case series, invasive micropapillary carcinoma, mucinous micropapillary carcinoma of breast, pure mucinous carcinoma, rare breast cancer

## Abstract

**Introduction and Importance:**

Mucinous micropapillary carcinoma breast (MMPC) is one of the rarest subtypes of breast cancer (BC) with clinicopathologic features correlated with poor prognosis. We are aimed at identifying MMPC cases and correlate them with histopathological features, overall survival (OS), and recurrence‐free survival (RFS).

**Case Presentation/Materials and Methods:**

We retrospectively identified cases of MMPC from our institutional repository from 2017 to 2022. Data were collected regarding age, stage of BC at the time of diagnosis, tumor size, lymphovascular invasion (LVI), extranodal extension (ENE), lymph node (LN) metastasis, tumor biology, recurrence, and OS. Quantitative variables are calculated as median with range (IQR), whereas qualitative variables are presented in percentages.

**Clinical Discussion/Results:**

Nine MMPC cases were reported in the last 6 years, with a median age at diagnosis of 49 years. At diagnosis, 66.67% (6/9) were Stage III followed by Stage II; the average tumor size was 4.5 cm and the majority of tumors were Grade II (77.78%) followed by Grade III. LVI was seen in 66.67%, ENE in 33.33%, and LN metastases in 66.67%. Estrogen receptor (ER)/progesterone receptor (PR) was positive in 77.78%. The OS was 7/9 (77.78%) at the median follow‐up of 3.5 years; recurrence was reported in two patients with an RFS of 77.78% (7/9).

**Conclusion:**

Our results demonstrate that MMPC is a rare entity that presented at a younger age with more advanced disease. Patients presented with larger tumor size, intermediate‐ to high‐grade tumor morphology, LVI, ENE, and LN metastasis. These tumors also overexpressed ER and PR. Despite this, clinical outcomes in our cohort were relatively favorable, which is also reflected in OS (77.78%) and RFS (77.78%) at a median follow‐up of 3.5 years.

## 1. Introduction

Mucinous carcinoma (MC) of the breast is a rare subtype of breast cancer (BC), accounting for about 2% of all cases. It is classified as a special type with a predilection for older populations (> 55 years) and is generally associated with a favorable prognosis, low recurrence rates, and low incidence of lymph node (LN) metastasis [1].

**Figure 1:**
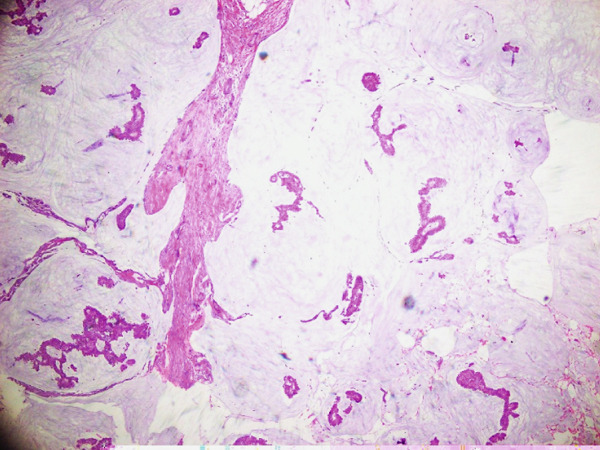
H & E stain showing mucinous carcinoma breast (10×) small uniformed clusters of cells floating in large pools of extracellular mucin.

**Figure 2:**
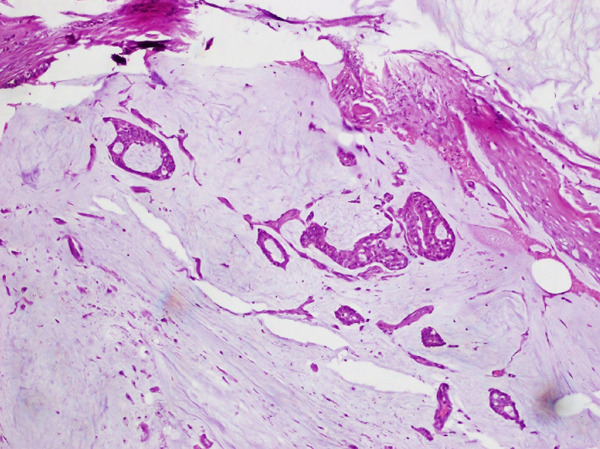
H & E stain mucinous carcinoma breast (20×) epithelial clusters floating in large extracellular mucin pools.

World Health Organization (WHO) defines MC as an entity composed of small uniform clusters of tumor cells floating in large pools of extracellular mucin [2], (Figures 1 and 2). National Comprehensive Cancer Network (NCCN) also enlisted it among BC with favorable prognosis. The subsequent management and decision to administer chemotherapy varies with the diagnosis of pure mucinous carcinoma (PMC) [2].

**Table 1:** This table compares different studies with our cohort of the patients.

	Peng Sun et al. *n*‐32	Y Sun et al. *n*‐40	Kim HJ *n*‐17	Our cohort *n*‐9
Laterality	—	—	—	Right
Median age	42 years	57 years	53 years	49 years
T size	—	2 cm (55.0%)	41.2%	4.5 cm (T3)
Stage	—	Stage I/II, 62.5%	—	111 (66.67%)
Tumor grade	Higher	—	Higher (82.4%)	Higher (77.8%)
LVI	Frequent	30.0%	—	(66.7%)
LN metastasis	Frequent	37.5%	23.5%	(66.7%)
ENE	—	—	—	33.3%
ER/PR status	No significant difference	85.0%	41.2%	77.78%
Her2 neu status	28.1%	12.5%	0%	11.11%
Ki67	31%	25.0%	58.8%	—
Follow‐up	Median, 52 months	76 months	83 months	42 months
OS	—	78%	—	77.78%
RFS	Decreased	—	—	77.78%

**Figure 3:**
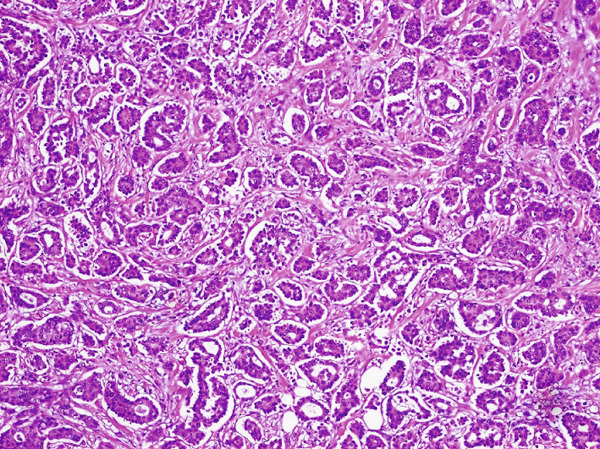
Micropapillary carcinoma breast on H & E stain (10×) small solid clusters of cells without fibrovascular cores with retraction artifacts.

**Figure 4:**
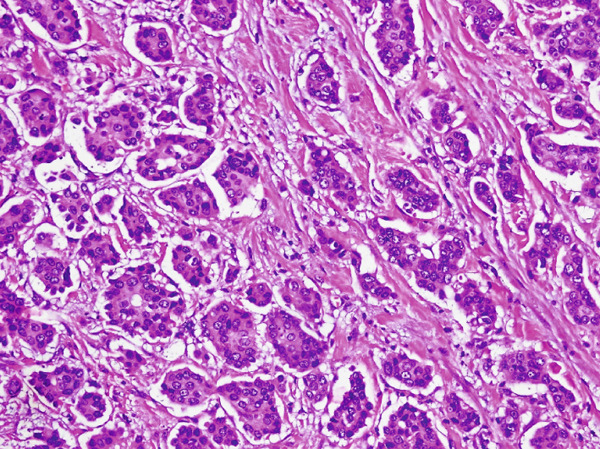
Micropapillary carcinoma breast on H & E stain (20×) small solid clusters of cells without fibrovascular cores with retraction artifacts.

At the other end of the spectrum is invasive micropapillary carcinoma (IMPC), which accounts for 1%–6% of BC and has a clinically aggressive course. It has strong predilection for lymphovascular invasion (LVI), LN metastasis and poorer outcomes [3]. Histologically, it is characterized by small, solid clusters of tumor cells arranged in a micropapillary, ring‐like structures (Figures 3 and 4), often highlighted by and better appreciated via immunohistochemistry (IHC) stain of epithelial membrane antigen (EMA) or Mucin‐1. It would show its characteristic inside–out pattern. Majority of this cohort of patients require aggressive management with the inclusion of chemotherapy [3].

**Figure 5:**
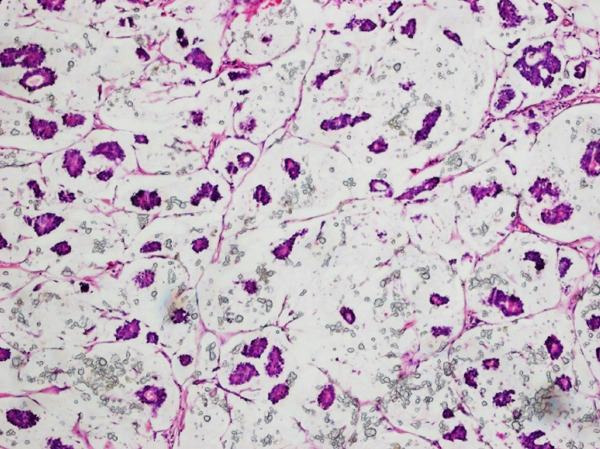
Mucinous micropapillary carcinoma breast on H & E stain (10×) micropapillary features in the background of mucinous carcinoma.

**Figure 6:**
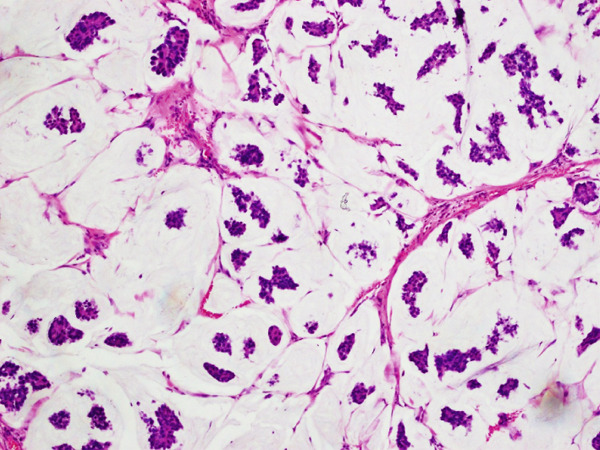
Mucinous micropapillary carcinoma breast on H & E stain (20×) micropapillary clusters lying in a mucinous background.

**Figure 7:**
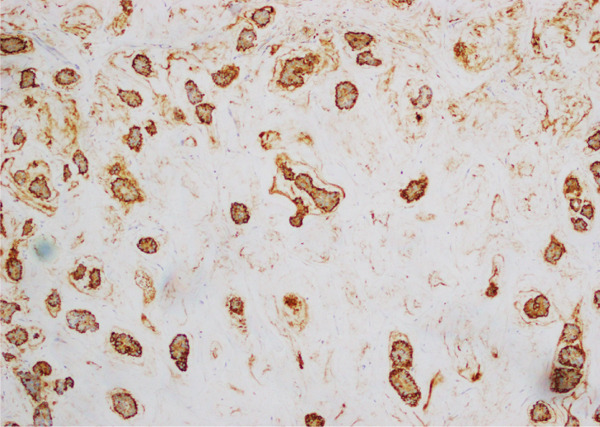
Photomicrograph showing EMA stain in mucinous micropapillary carcinoma. Cellular outlines are highlighted by brown stain emphasizing reverse polarity of cells.

**Figure 8:**
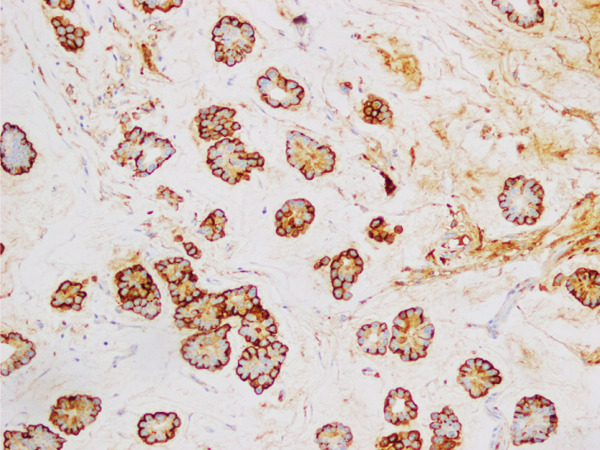
Photomicrograph showing a closer look (20×) of EMA stain in mucinous micropapillary carcinoma.

Between these two extremes lies the much rarer “mucinous micropapillary carcinoma” (MMPC), which according to literature accounts for less than 1% of BC cases [4]. On gross examination, these tumors are lobulated with mucin evident on the cut section. Histologically, they combine features of MC and IMPC. These tumors show micropapillary clusters, hobnailing, occasional psammomatous calcifications, and characteristic reverse polarity in pools of extracellular mucin [5, 6] The nuclear grade of the tumor is usually intermediate to high [5, 6], (Figures 5 and 6). IHC is largely not required; however, EMA still can highlight the inside–out arrangement of tumor cell clusters (Figures 7 and 8).

**Figure 9:**
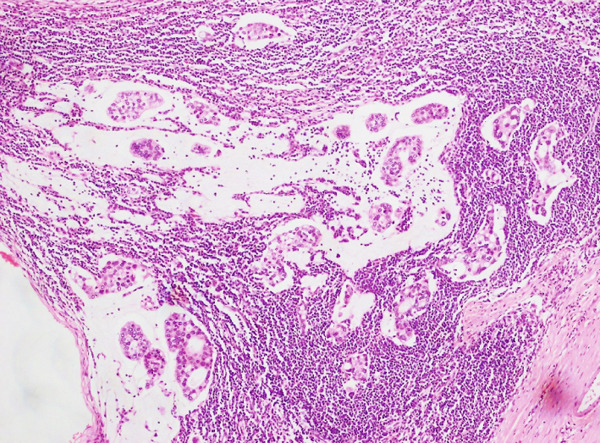
H & E stain showing mucinous micropapillary carcinoma breast (10×) exhibiting lymph node metastasis.

The clinical–pathological features of this hybrid subtype are yet to be discovered. We have limited data, and studies show conflicting results. The majority of the literature favors MMPC, demonstrating at a younger age, higher nuclear grade, and increased propensity for LVI and LN metastasis (Figure 9). These features make the further management of patients with MMPC questionable; as to whether these patients need aggressive management or not [5–7].

Rarity of MMPC and uncertainty regarding its behavior and prognosis prompted us to study and assess its clinicopathological features presenting to our center. We are aimed at correlating tumor features with the clinical stage, overall survival (OS), and recurrence‐free survival (RFS). This will help us stratify MMPC patients at the time of initial diagnosis and address the disease more confidently.

## 2. Materials And Methods

Patients with histopathologically proven MMPC were identified from retrospectively maintained data of the histopathology section of the pathology department at our hospital from the last 6 years that is, Jan. 2017–Dec. 2022. Using ICD coding, MMPC cases were extracted from hospital records, and data were collected regarding age at diagnosis, tumor laterality, stage of BC at diagnosis, recurrence, and OS status from hospital records and the last visits of the patients reported in the system. The histopathological characteristics like tumor size, LVI, extranodal extension (ENE), LN metastasis, and tumor biology were also recorded for each patient. Standard surgical, chemotherapy, and radiation treatments were provided according to the institutional tumor stage and biology protocols. Quantitative age, tumor size, and OS variables are calculated as median with range (IQR), whereas qualitative variables are presented in percentages and frequencies. The research work has been reported in line with the PROCESS guidelines [8, 9].

## 3. Results

In the last 6 years, nine cases have been reported to our center with biopsy‐proven MMPC. The median age of BC diagnosis in our patients was 49 years with an age range between 27–74 years (IQR: 24 years). According to laterality, cancers were reported more on the right breast 5/9 (55.56%) than on the left side 4/9 (44.44%). At the time of diagnosis, 66.67% (6/9) of patients were diagnosed with Stage III, whereas 33.33% (3/9) were diagnosed with Stage II. Regarding histopathological features, the average reported tumor size in our cohort of cases was 4.5 cm with a range of 2.5–7 cm (*I* *Q* *R* = 2.9 *c* *m*). The majority of tumors were Grade II, 77.78% (7/9) at the time of diagnosis; followed by Grade III, 22.22% (2/9). LVI was seen in 6/9 (66.67%) patients and ENE was appreciated in 3/9 that is, 33.33% of patients. LN metastases were present in 6/9 (66.67%), with an average of four nodes identified. The maximum number of positive LNs seen was 17 in a patient. Regarding tumor biology, 77.78% of MMPC patients were estrogen receptor (ER) and progesterone receptor (PR) positive (7/9), whereas only one patient was Her 2 neu receptor positive (11.11%). At the median follow‐up period of 3.5 years (IQR: 42 months or 3.5 years), recurrence was reported in two patients (22.22%). Both patients were initially diagnosed at Stage III, and one had Her 2 Neu positive status along with ER/PR positivity. Therefore, the RFS reported in our cohort is 77.78% (7/9). The OS reported at the time of the last data entry is seven out of nine patients (77.78%). The two patients who did not survive experienced distant metastasis of the disease/recurrence.

## 4. Discussion

Due to the rarity of MMPC in our clinical practice, that is around 0.1%–0.3% of total BC cases, we have limited data and information regarding its behavior and clinicopathological features. There is a high chance of misclassification of MMPC, which has implications on prognosis and treatment strategies [10]. The identification of just nine cases over 6 years′ time of the study further emphasizes its rarity. Our study, which was aimed at understanding the biological behavior, found out that the MMPC has predilection for younger age group (median age: 49 years) [7, 10, 11] with a slight tendency towards right side, 55.56%. This was also predicted in another study which showed 56.2% of right sided disease [11].

At presentation, the majority of patients (66.7%) had Stage III disease, and the average tumor size was 4.5 cm, T3. Histopathologically, most tumors were Grade II (77.8%), with high rates of LVI (66.7%), ENE (33.3%), and LN metastases (66.7%). Despite these high‐risk features, the observed clinical outcomes were relatively favorable.

If we compare our results with other sources, a study mentioned the majority of their patients having Stage 2 disease (47%), followed by Stage 1 BC. The variation between our results (i.e., 66.7% of patients having Stage III disease) could be due to differences in geographical regions in which research was carried out. High‐income nations have better screening options for their population which helps in identifying BC at the initial stages [10]. Similarly, the majority of patients in our cohort were T3, to begin with, which is in contrast to another research which showed T2 being the dominant pattern [11]. This study also states comparable results with respect to tumor grade and LVI [11].

Presence of extensive LN metastasis in our cohort (66.67%) emphasizes its aggressive nature as compared with LN metastasis in PMCs [10–13]. Having said this, it is important to know that LN metastasis directly correlates with the tumor size, which was already high in our data probably due to the late presentation of our patients in general.

Studies have also looked into molecular profiling at the cytogenetic level. They have inferred the importance of PIK3CA, GATA3, TP53, SF3B1, mTOR, and ErbB mutations along with the interplay of 1q, 6p, 8q, 10q, 16q, 11q, and 13q at the genetic level. This makes us more certain that MMPC is an overlapping entity between PMC and IMPC [11, 12]. It tells us that perhaps this newly acknowledged group of tumors is heterogeneous at the molecular level and may not make up one morphological type, but rather a convergent phenotype. GATA3, TP53, and SF3B1 are repeatedly seen to be mutated in MMPCs. MMPCs also display 17q, 20q gains, and 17p losses [10].

The significance of high ki‐67 levels is also reported in many studies which helps us in understanding its aggressive nature. We were unable to study these parameters in our cohort due to institutional limitations [10, 11].

Talking about hormone status and Her 2 Neu expression in our study, our results were comparable with other studies showing ER/PR predominance among their patients [5, 11, 12]. Although one of our patients had Her 2 Neu amplification detected who also had disease recurrence as Stage IV, it coincides with the predicted bad prognosis among MMPC patients who overexpress Her 2 Neu gene [10, 12, 14].

The fact that MMPC were ER/PR positive and Her2 neu negative and still possessed aggressive clinical and pathological features can mainly be assigned to tumor biology that is, presence of micropapillary component, which is known to have aggressive nature. Other than this, late presentation, poor understanding and deficient breast screening program has also contributed to increased tumor size, LVI, LN metastasis, and ENE.

The OS and RFS reported in our cohort are 77.78%, respectively, with a median follow‐up period of 3.5 years. One study reported that MMPC is associated with decreased OS and RFS compared with PMC, whereas when compared with IMPC, it has better OS and RFS [15]. Another study reported a favorable prognosis when compared with its counterparts, so the literature is showing mixed results for MMPC [12, 13]. Therefore, experts suggest that while considering special tumor subtypes, clinicians should take other prognostic factors like T size, LN status, and tumor biology into account to stratify the patients and devise management plans [16].

Our study has several limitations that must be acknowledged. Firstly, the small sample size limits the generalizability of our findings and may not represent the broader population of patients with MMPC. Secondly, the retrospective nature of the study relies on existing medical records, which may be incomplete or subject to reporting bias. We were also unable to perform detailed molecular profiling or assess ki‐67 levels due to institutional limitations, which could have provided deeper insights into tumor biology and potential prognostic factors. Lastly, the variation in diagnostic stages and treatment protocols over the study period could introduce heterogeneity in patient outcomes, making it difficult to draw definitive conclusions. Future studies with larger cohorts and prospective designs are needed to validate our findings and explore the molecular characteristics of MMPC thoroughly.

## 5. Conclusion

Our study highlights the rarity and aggressive nature of MMPC, which presents at a younger median age of 49 years with advanced stage and larger tumor size. Histopathological features include high‐grade tumor, frequent LVI, significant LN metastasis, and ENE. Despite these aggressive characteristics, the OS and RFS rates were 77.78% over a median follow‐up of 3.5 years.

These findings emphasize the need for early detection and tailored management strategies for MMPC. Future research with larger cohorts is necessary to validate our results and further understand MMPC′s molecular characteristics. In the developing world, enhancing BC awareness and screening programs is crucial to improve early diagnosis and outcomes for patients with this rare subtype.

**Nomenclature**
MMPC: mucinous micropapillary carcinoma breast
BC: breast cancer
OS: overall survival
RFS: recurrence‐free survival
LVI: lymphovascular invasion
ENE: extranodal extension
LN: lymph node
ER: estrogen receptor
PR: progesterone receptor
NCCN: National Comprehensive Cancer Network
PMC: pure mucinous carcinoma
IMPC: invasive micropapillary carcinoma
IHC: immunohistochemistry
EMA: epithelial membrane antigen

## Funding

No funding was received for this manuscript.

## Ethics Statement

The authors have nothing to report.

## Consent

Written informed consent was obtained from the patient for publication of this case series.

## Conflicts of Interest

The authors declare no conflicts of interest.

## Data Availability

The data that support the findings of this study are available on request from the corresponding author. The data are not publicly available due to privacy or ethical restrictions.
